# 

**Published:** 1891-03

**Authors:** 


### PENNSYLVANIA STATE VETERINARY MEDICAL ASSOCIATION.

The annual meeting of the Pennsylvania State Veterinary Medical Association. will be held on Tuesday, March 3, 1891, at the College of Physicians, 13th and Locust Streets., Philadelphia.

The meeting will be called to order at ten o'clock A. M. A full attendance from all sections of the State is expected as in addition to the technical papers which will be presented, the important subject of amendment of the Act Regulating Veterinary Practice will be discussed.

